# Significance of Dufour and Soret aspects on dynamics of water based ternary hybrid nanofluid flow in a 3D computational domain

**DOI:** 10.1038/s41598-023-30609-9

**Published:** 2023-03-14

**Authors:** Sardar Bilal, Muhammad Imran Asjad, Shams ul Haq, Musawa Yahya Almusawa, ElSayed M. Tag-ElDin, Farhat Ali

**Affiliations:** 1 Department of Mathematics, Air University, Sector E-9, P.A. F Complex, Islamabad, 44000 Pakistan; 2 Department of Mathematics, University of Management and Technology, Lahore, 54770 Pakistan; 3 Department of Mathematics, Faculty of Science, Jazan University, Jazan, 45142 Saudi Arabia; 4 Faculty of Engineering and Technology, Future University in Egypt, New Cairo, 11835 Egypt; 5 Department of Architecture and Planning, College of Engineering, King Khalid University, Abha, Saudi Arabia

**Keywords:** Engineering, Materials science, Mathematics and computing

## Abstract

The prime motive to conduct this communication is to explicate hydrothermal attributes of water by inducing new composition of nanoparticles termed as ternary particles. For this purpose, two differently natured groups one with lesser densities (Carbon nanotubes, Graphene and Aluminium oxide) and with higher densities (Copper oxide, Copper and Silver) are accounted. A 3D permeable surface is considered as a physical configuration of problem by providing dual stretching. Initially, mathematical structuring in dimensional representation expressing the constitutive relations for mass, momentum and energy conservation is manifested. Later on, a set of similar variables are executed to express attained coupled system into ordinary form. Numerical simulations are performed to find solution by employing shooting and RK-4 methods in conjunction. Description about change is displayed through graphical visualization. Subsequently, temperature distribution and heat flux coefficient against sundry variables are also measured and comprehensively discussed in pictorial and tabular format. Wall drag coefficients along (x, y) directions are also computed. It is inferred from the outcomes that velocity, temperature and concentration of base fluid is higher for ternary group 1 containing particles of low densities than for group 2 with more denser particles. It is also deduced that elevation in temperature of fluid is revealed against Soret number whereas contrary aspects is observed in view of concentration distribution. Dufour number has declining impact on temperature profile whereas it upsurges the mass distribution. It is depicted that skin friction in case of group containing particles with less densities are more than other group.

## Introduction

Nanoliquid is the colloidal composition of nanometric size particles and ordinary liquids. These particles are induced to enhance thermal features of ordinary liquids which possesses low thermal conductivities. Incorporation of these engineered particles have raised their applicability in numerous procedures like in, thermal management in vehicles, uplift and reduction in temperature, microelectronics, pharmaceutical procedures and so forth. After attaining deep knowledge and experimentations about intensive composition of nanoparticles and base liquids the idea of addition of two differently natured nano composition has persuaded known as hybrid nanofluid. Hybrid nanofluids are employed in multiple procedures like in solar energy, blast and air chilling applications, electronic freezing, automatic industry, generator cooling, transformer cooling, nuclear process, biomedical engineering and so forth. Presently many researchers have tried to studies on hybrid nano liquids. Since, the knowledge of utilization of hybrid nanofluids is basically to boost thermophysical properties. So, hybrid nanofluids are built-up as a novel class produced by merging two nanoparticles containing metal or metal oxides as a combination. It is experimentally proved that hybrid nanofluids show promising elevation in thermophysical properties as related with mono nanofluids especially in the case of thermal conductivity. Suresh et al.^1^ initiated the thought of hybrid nanofluids to boost the heat transfer rate of simple fluid this concept gave a new field to researchers to do work in this direction. Afterwards, several thought-provoking research has been conducted in recent years like, Bhattad^2^ experimentally examined the exergy and energy features of Al_2_O_3_–MgO hybrid nanofluids by mixing with different base liquids in a heat exchanger. Phanindra et al.^3^ induced Al_2_O_3_–Cu hybrid nanoparticles to analyze heat transfer in a concentric tabular exchanger filled with oil and found 12.06% boost in thermal conductivity. Abiodun et al.^4^ delineated minimization of entropy generation to improve thermal efficiency of viscous hybrid nanofluid defined by Eyring–Powell model. They accounted Cu–Al_2_O_3_ hybrid nanoparticles in ethylene glycol and analyzed the aspects of nanoparticles volume fractions. Taza et al.^5^ scrutinized flow of water-based carbon nanotubes of hybrid nanofluids. Liaqat et al.^6^ contemplated hybrid nanofluid transport mechanism in a rotating frame by incorporating single phase nano model along with induction of Al_2_O_3_ and Cu nanoparticles. Hayat et al.^7^ demonstrated improvement in ordinary base liquid by adding hybrid nanoliquid in the presence of momentum and thermal slip. They also assumed reduction in the heat transmission in the flow and performed numerical simulations by exactly numerical scheme. Mabood et al.^8^ explicated fully developed forced convective flow of water-based hybrid nanofluid generated over a stretched surface in the existence of thermal radiation and melting heat transfer. Amir et al.^9^ explored heat transmission and flow in a passage full with Cu–Al_2_O_3_ hybrid nanofluid with provision of heat flux and viscous dissipation effect. Li et al.^10^ performed computational scheme to access hydrothermal performance and irreversibility phenomenon of water based hybrid nanofluid induced with Mgo–Ag particles filled in sinusoidal hairpin shaped heat exchanger. Benkhedda et al.^11^ studied mixed convection in horizontal annulus filled with a TiO_2_/water nanofluid and seeing Ag–TiO_2_/water hybrid nanofluid providing uniform heating at outer cylinder and inner cylinder adiabatic. Hassan et al.^12^ dealt with flow and convective thermal performance of Cu–Ag/water hybrid nanofluid over an inverted cone. Boussinesq and boundary layer approximations are capatilized to model governing equations. Analytical solutions are obtained to adumbrate aspects of nanoparticles volume fraction and hybrid nanoparticles on velocity and temperature distributions. Rahman et al.^13^ manifested thermal behavior of hybrid nanofluids comprising of Al_2_O_3_ and Cu nanoparticles induced in 2D axisymmetric copper tube. Saeed et al.^14^ performed a comprehensive study about flow of hybrid nanofluid synthesized by cylindrical shaped carbon nanotubes (SWCNTs and MWCNTs) and Fe_3_O_4_ over an exponentially spreading bowed surface embedded in permeable medium. Nadeem et al.^15^ explored stagnant flow of 3D hybrid nanofluid past a circular cylinder in the existence of thermal slip effects. They noticed uplift in heat transfer rate in case of addition of hybrid nanoparticles as compared to absence of particles in base liquid. Wei et al.^16^ experimentally measured sedimentation process in newly fabricated hybrid nanofluid comprising of oil based TiO_2_ fluid and measured appreciable uprise in thermal conductivity of base-liquid. Sundar et al.^17^ observed the dynamics of hybrid nanofluid synthesized by addition of ethylene glycol nanoparticles in water. Chamkha et al.^18^ inquired magnetically effected flow of hybrid nanofluid in a rotating system between two penetrable and stretchable surfaces. Momin^19^ carried out study on mixed convective heat transfer in water with addition of Al_2_O_3_ hybrid particles flowing over an induced surface. Ashwinkumar^20^ presented analytical study on magnetized flow of Al_50_Cu_50_/Ag water nanofluid over a stretched surface with thermal diffusion effects. From attained results he portrayed that thermal diffusion transport augments with the addition of hybrid nanoparticles. Sulochana and Ashwinkumar^21^ reported the stimulus of thermophoresis and Brownian movement on 2D magnetized forced convection flow of nanofluid past a permeable elongating surface. Mabood et al.^22^ scrutinized simultaneous effects of 2D unsteady hybrid nanofluid over a flat surface with radiative heat flux. They discussed that uplift in nanoparticles volume triggers the thermofluidic characteristics of base fluid. Mabood et al.^23^ performed simulations for stagnant flow of hybrid nanoliquid composed of Fe_3_O_4_ and graphene in water. They noticed enhancement in local heat flux with addition of these hybrid particles instead of consideration of only base fluid. The steady 2-D mixed convective stagnant point flow of hybrid nanofluid over vertical plate with radiation, Dufour and Soret impacts was explored by Wahid et al.^24^. The flow of hybrid nanofluid over permeable stretching sheet under the appliance of magnetohydrodynamics (MHD) and radiations effect was numerically explored by Syahirah et al.^25^. Wahid et al.^26^ made the numerical analysis of magneto nanofluid in three dimensions past over shrinking surface with suction and thermal radiation impacts.

More recently, the dominance of the hybrid nanofluid over the different nanofluids has been confirmed. On basis of above mentioned theoretical and experimental studies researches are focusing on colloidal combination of three different types of nanoparticles with in single base fluid. The resultant fluid has numerous names, like trihybrid nanofluids, ternary hybrid nanofluids and ternary nanofluid. Applications of ternary carbonate nanofluid and carbonate nanotubes are found in supercritical solar power plants due to its constancy and effected thermal diffusivity^27^. Mousavi et al.^28^ elucidated the ternary hybrid nanofluid flow of water containing copper oxide, magnesium oxide and titanium oxide and concluded that viscosity of ternary mixture uplift with nanoparticle volume fraction down surges with temperature. Sahoo et al.^29^ addressed boost in specific heat capacity of base fluid with addition of ternary hybrid nanofluid containing aluminum, copper oxide and titanium oxide. The influence of endothermic/exothermic chemical reactions along with activation energy on a ternary hybrid nanofluid with the wedge geometry is taken into account by Sajid et al.^30^. The effect of Diathermic oil (DO) with the inclusion of tri-hybrid nanoparticles by adopting a tri-hybrid Yamada-Ota and Xue nanofluid model was explained by Sajid et al.^31^. Elnaqeeb et al.^32^ deliberated the interruption of carbon nanotubes, graphene and aluminum in water along with the affection of various particles shapes and size. They concluded that temperature enhances at small volume fraction.

Communication of heat and mass in a moving fluid yielded by mass and energy fluxes is a renowned as Dufour and Soret aspects. Consideration of energy gradients phenomenon especially in convective flow transport mechanism possesses noteworthy importance like in cooling applications, steel industries and heat exchangers. In addition, Dufour and Soret diffusion phenomenon plays vital role in designing of nuclear reactors, geo thermal energy, migration of pollutants, and departure of elements, gases mixture formation and nuclear waste disposal. Some salient research efforts on heat and mass transfer in existence of Dufour and Soret aspects can be seen here by. Hayat et al.^33^ manipulated Soret and Dufour aspects in magnetically effected flow of Casson fluid over a stretched surface by finding analytical solution. Pal and Mondal^34^ analyzed joint influence of Dufour and Soret effects on mixed convective heat transfer over a stretched surface. They found that temperature profile increases against Dufour number and concentration field also elevates versus Soret number. Hayat et al.^35^ discussed flow transport in tangent hyperbolic liquid with Soret and Dufour diffusion. They observed that inclusion of these effects combinedly enhance the temperature and concentration fields. Shojaei et al.^36^ performed hydrothermal analysis of non-Newtonian rate type of fluid flow over a radiative stretching cylinder in existence of Soret and Dufour phenomenon. It is premediated that coincident variation of Dufour and Soret has an inverse relation with mass transfer rate. Jawad et al.^37^ discussed transport procedure in magnetized flow of Maxwell fluid with thermo diffusion aspects. They found that thermo diffusion phenomenon is more effective that Brownian diffusion due to generation of temperature and concentration gradient. Ramzan et al.^38^ studied 3D chemically reactive upper-convicted flow over a stretching surface in the existence of Dufour and Soret aspects. Ramzan et al.^39^ worked on thermal diffusion on mixed convective boundary layer flow of viscoelastic nanofluid over a vertical stretching surface surrounded in a leaky medium. Kabeir et al.^40^ worked on heat and mass transfer in magnetized flow of mixed convective stagnant power law non-Newtonian fluid with diffusion aspects. Dufour and Soret impacts on magnetohydrodynamics mixed convective fluid flow of cross fluid was examined by Rehman et al.^41^. Shahnaz et al.^42^ made the mathematical modeling for 2-D double diffusive flow of MHD Maxwell fluid. The impacts of magnetic field accompanied with Dufour and Soret on 3D cross fluid flow in the streamwise direction was explored by Khan et al.^43^. Shahzad et al.^44^ analyzed the MHD flow of hybrid nanofluid conventional nanofluid between two plates. Exponentially stretched sheet caused fluid flow. The flow properties and heat transfer that occurs between hybridized nanofluid and solar system was studied by Mohamed^45^. The heat transfer of MHD radiative Carreau hybrid nanofluid was analyzed by Ahmed and Mohamed^46^.

Scrutinization about hydrodynamical characteristics of flow transpiration over a surface with provision of suction/injection is a noteworthy process in fluid dynamics. Efficiency of working of washing machines is one of exemplary process based on the concept of suction/injection. In addition, sedimental transport and separation of contaminants for controlling healthy ecosystem is also based on suction/injection phenomenon. In flow domain Gregory and Walker^47^ performed experimentation to control rapid turbulence by raising the rate of suction of fluid from surface. Smyth et al.^48^ measured spreading of energy during the suction and boosting of arteriovenous pressure difference for fluid flow in vessels. Krogmann^49^ presented worthy remark about suction in boundary layer flows for controlling boundary layer separation between fluid and solid surfaces. Hayat et al.^50^ studied flow of Jeffery fluid in leaky passage under the appliance of magnetic field.

Electrically conducting fluids which get ionization on interaction with magnetic field are involved in many metallurgical processes like cooling of incessant strips, sketch and annealing of copper wires, refinement of melted wastes from non-metallic inclusions and so forth. The primary effort, to the author’s knowledge to investigate MHD flow over an elongating surface was persuaded by Pavlov^51^. In recent years due to advancement in technology magnetized are used in multiple procedures. So, researcher has found the applications of these particles in cell parting, affecting of drugs, magnetic resonance imaging (MRI) and so forth. Sandeep^52^ investigated flow and heat transfer aspects of magnetic–nanofluid over a stretching surface with non-uniform source and thermal radiation. Ahmed and Nadeem^53^ examined the effect of metallic hybrid nanoparticles on flow of viscous fluid over a stretched surface. Zaib et al.^54^ deliberated that the magnetic nanofluids are used in cancer therapeutics and imaging. Furthermore, the nanoparticles of this type are also used to destroy cancerous cells. Wakif^55^ carried out study on MHD convective variable flow of Casson viscoelastic liquid under influence of thermal radiations with viscosity and thermal conductivity.

From existing studies, it is evaluated that, Dufour and Soret effects on 3D flow of water-based hybrid nanofluid by inserting ternary hybrid nano composition of low and higher densities namely ternary group 1 (Carbon nanotube, Graphene and Aluminium oxide) and ternary group 2 (Copper oxide, Copper and Silver) through a three-dimensional computation fastened domain with significant focus on suction and dual stretching has not yet been interrogated.

Arrangement of conducted analysis is summarized in following manner. Firstly, overview of studies conducted by different researchers is reviewed afterwards formulation of problem representing physics of problem is presented. After then, description about implemented numerical scheme is discussed and by using it results are drawn and analyzed. At last, key findings are enumerated to assist researchers working in this direction.

## Mathematical formulation

**Figure 1:**
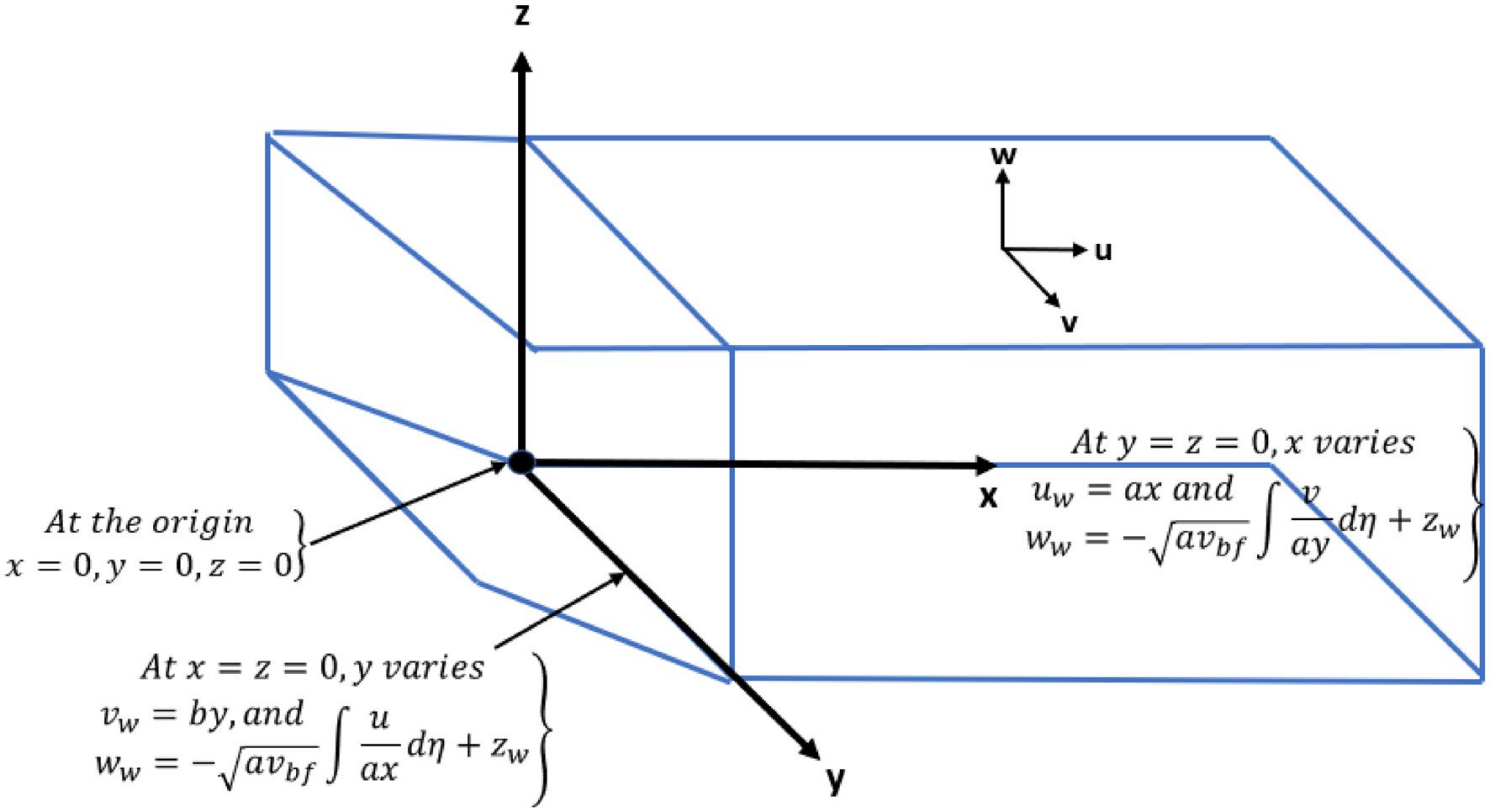
Physical configuration of problem.

We have assumed 3D steady flow of viscous (water) fluid over a dually stretched surface with suction aspects. Ternary nanoparticles of low and high densities are added in the base fluid along with considering different shapes of particles as shown in Fig. 1.

The governing mass, momentum, energy and concentration equations are uttered below^56^

1 $$\frac{\partial u}{{\partial x}} + \frac{\partial v}{{\partial y}} + \frac{\partial w}{{\partial z}} = 0,$$

2 $$u\frac{\partial u}{\partial x}+v\frac{\partial u}{\partial y}+w\frac{\partial u}{\partial z}=\frac{{\mu }_{hnf}}{{\rho }_{hnf}}\frac{{\partial }^{2}u}{\partial {z}^{2}},$$

3 $$u\frac{\partial v}{\partial x}+v\frac{\partial v}{\partial y}+w\frac{\partial v}{\partial z}=\frac{{\mu }_{hnf}}{{\rho }_{hnf}}\frac{{\partial }^{2}v}{\partial {z}^{2}},$$

4 $$u\frac{\partial T}{\partial x}+v\frac{\partial T}{\partial y}+w\frac{\partial T}{\partial z}=\frac{{k}_{hnf}}{{{(\rho }_{cp})}_{hnf}}\frac{{\partial }^{2}T}{\partial {z}^{2}}+\frac{D{K}_{T}}{{C}_{s}{C}_{p}}\frac{{\partial }^{2}C}{\partial {z}^{2}},$$

5 $$u\frac{\partial C}{\partial \mathrm{x}}+v\frac{\partial C}{\partial \mathrm{y}}+w\frac{\partial C}{\partial \mathrm{z}}=D\frac{{\partial }^{2}C}{\partial {z}^{2}}-{K}_{1}C+\frac{D{K}_{T}}{{T}_{m}}\frac{{\partial }^{2}T}{\partial {z}^{2}}.$$

Boundary conditions of (Momentum, Temperature and Concentration) in all directions are described below

6 $$u = u_{w} \left( x \right) = ax,w = w_{w} \left( { - \sqrt {a\vartheta _{{bf}} \int {\frac{v}{{ay}}} d\eta + z_{w} } } \right)\;{\text{at}}\;z = 0,\;{\text{and}}\;y = 0,u \to 0\;{\text{as}}\;z \to \infty \;{\text{and}}\;y = 0.$$

7 $$v = v_{w} \left( x \right) = by,w = w_{w} \left( { - \sqrt {a\vartheta _{{bf}} \int {\frac{u}{{ax}}} d\eta + z_{w} } } \right)\;{\text{at}}\;z = 0\;{\text{and}}\;x = 0,v \to 0,\;{\text{as}}\;z \to \infty \;{\text{and}}\;x = 0.$$

Boundary conditions for Eqs. (4) and (5)

8 $$T = T_{w} ,C = C_{w} ,\;{\text{at}}\;z = 0,T \to T_{\infty } ,C \to C_{\infty } ,\;{\text{as}}\;z \to \infty .$$

Following Transformations used to transform dimensional equations into dimensionless equations

9 $$\eta =z\sqrt{\frac{a}{{\vartheta }_{bf}}}, u=ax\frac{df}{d\eta }, v=by\frac{dg}{d\eta }, w=-\sqrt{a{\vartheta }_{bf}\left[f\left(\eta \right)+cg\left(\eta \right)\right]}, c=\frac{b}{a}, {f}_{w}=\frac{-{z}_{w}}{\sqrt{a{\vartheta }_{bf}}}, \theta =\frac{T-{T}_{\infty }}{{T}_{w}-{T}_{\infty }}, \varphi =\frac{C-{C}_{\infty }}{{C}_{w}-{C}_{\infty }},Df=\frac{{Dk}_{T}}{{C}_{s}{C}_{p}}\frac{\left({C}_{w}-{C}_{\infty }\right)}{\left({T}_{w}-{T}_{\infty }\right)\vartheta },Sr=\frac{{Dk}_{T}}{{T}_{m}\vartheta }\frac{\left({T}_{w}-{T}_{\infty }\right)}{\left({C}_{w}-{C}_{\infty }\right)},\gamma =\frac{{K}_{1}}{a}.$$

Thermophysical relations for viscosity, thermal conductivity, density and heat capacitance of hybrid nanofluid are given as under

10 $${\mu }_{hnf}=\frac{{\mu }_{nf1}{\phi }_{1}+{{\mu }_{nf2}\phi }_{2}+{\mu }_{nf3}{\phi }_{3}}{{\left(1-\phi \right)}^{2.5}}, {k}_{hnf}=\frac{{k}_{nf1}{\phi }_{1}+{k}_{nf2}{\phi }_{2}+{k}_{nf3}{\phi }_{3}}{{(1-\phi )}^{2.5}}.$$

11 $${\rho }_{hnf}=\left(1-{\phi }_{1}-{\phi }_{2}-{\phi }_{3}\right){\rho }_{bf}+{\phi }_{1}{\rho }_{sp1}+{\phi }_{2}{\rho }_{sp2}+{\phi }_{3}{\rho }_{sp3}.$$

12 $${(\rho {c}_{p})}_{hnf}=\left(1-{\phi }_{1}-{\phi }_{2}-{\phi }_{3}\right){{(\rho c}_{p})}_{bf}+{\phi }_{1}{\left(\rho {c}_{p}\right)}_{sp1}+{\phi }_{2}{\left(\rho {c}_{p}\right)}_{sp2}+{\phi }_{3}{\left(\rho {c}_{p}\right)}_{sp3}.$$

The viscosity and thermal conductivity models for spherical, cylindrical and platelet nanoparticles are as under

13 $$\frac{{\mu }_{nf1}}{{\mu }_{bf}}=1+2.5\phi +6.2{\phi }^{2}, {k}_{nf1}={k}_{bf}\left[\frac{{k}_{sp1}+{2k}_{bf}-2\phi \left({k}_{bf}-{k}_{sp1}\right)}{{k}_{sp1}+{2k}_{bf}+\phi \left({k}_{bf}-{k}_{sp1}\right)}\right].$$

14 $$\frac{{\mu }_{nf2}}{{\mu }_{bf}}=1+13.5\phi +904.4{\phi }^{2}, {k}_{nf2}={k}_{bf}\left[\frac{{k}_{sp2}+3.9{k}_{bf}-3.9\phi ({k}_{bf}-{k}_{sp2})}{{k}_{sp2}+3.9{k}_{bf}+\phi ({k}_{bf}-{k}_{sp2})}\right] .$$

15 $$\frac{{\mu }_{nf3}}{{\mu }_{bf}}=1+37.1\phi +612.6{\phi }^{2}, {k}_{nf3}={k}_{bf}\left[\frac{{k}_{sp3}+{4.7k}_{bf}-4.7\phi ({k}_{bf}-{k}_{sp3})}{{k}_{sp3}+{4.7k}_{bf}+\phi ({k}_{bf}-{k}_{sp3})}\right] .$$

The accumulative volume fraction is represented as below

16 $$\phi ={\phi }_{1}+{\phi }_{2}+{\phi }_{3}.$$

Coefficient involved

17 $$\begin{aligned} & A_{2} = 1 - \left( {\phi _{1} + \phi _{2} + \phi _{3} } \right) + \phi _{1} \frac{{\rho _{{sp1}} }}{{\rho _{{bf}} }} + \phi _{2} \frac{{\rho _{{sp2}} }}{{\rho _{{bf}} }} + \phi _{3} \frac{{\rho _{{sp3}} }}{{\rho _{{bf}} }}, \\ & B_{1} = 1 + 2.5\phi + 6.2\phi ^{2} , \\ & B_{2} = 1 + 13.5\phi + 904.4\phi ^{2} , \\ & B_{3} = 1 + 37.1\phi + 612.6\phi ^{2} , \\ & B_{4} = \frac{{k_{{sp1}} + 2k_{{bf}} - 2\phi (k_{{bf}} - k_{{sp1}} )}}{{k_{{sp1}} + 2k_{{bf}} + \phi (k_{{bf}} - k_{{sp1}} )}}, \\ & B_{5} = \frac{{k_{{sp2}} + 3.9k_{{bf}} - 3.9\phi (k_{{bf}} - k_{{sp2}} )}}{{k_{{sp2}} + 3.9k_{{bf}} + \phi (k_{{bf}} - k_{{sp2}} )}}, \\ & B_{6} = \frac{{k_{{sp3}} + 4.7k_{{bf}} - 4.7\phi (k_{{bf}} - k_{{sp3}} )}}{{k_{{sp3}} + 4.7k_{{bf}} + \phi (k_{{bf}} - k_{{sp3}} )}}, \\ & A_{1} = B_{1} \phi _{1} + B_{2} \phi _{2} + B_{3} \phi _{3} , \\ & A_{4} = 1 - \left( {\phi _{1} + \phi _{2} + \phi _{3} } \right) + \phi _{1} \frac{{\left( {\rho c_{p} } \right)_{{sp1}} }}{{\left( {\rho c_{p} } \right)_{{bf}} }} + \phi _{2} \frac{{\left( {\rho c_{p} } \right)_{{sp2}} }}{{\left( {\rho c_{p} } \right)_{{bf}} }} + \phi _{3} \frac{{\left( {\rho c_{p} } \right)_{{sp3}} }}{{\left( {\rho c_{p} } \right)_{{bf}} }}, \\ & A_{3} = B_{4} \phi _{1} + B_{5} \phi _{2} + B_{6} \phi _{3} . \\ \end{aligned}$$

After using transformations the dimensionless form of equations are as under

18 $$\frac{{A}_{1}}{{A}_{2}{(1-\phi )}^{2.5}}\frac{{d}^{3}f}{d{\eta }^{3}}-\frac{df}{d\eta }\frac{df}{d\eta }+\left[f+cg\right]\frac{{d}^{2}f}{d{\eta }^{2}}=0,$$

19 $$\frac{{A}_{1}}{{A}_{2}{(1-\phi )}^{2.5}}\frac{{d}^{3}g}{d{\eta }^{3}}-c\frac{dg}{d\eta }\frac{dg}{d\eta }+[f+cg]\frac{{d}^{2}g}{d{\eta }^{2}}=0,$$

20 $$\frac{{A}_{3}}{{A}_{4}{(1-\phi )}^{2.5}}\frac{{d}^{2}\theta }{d{\eta }^{2}}+DfPr\frac{{d}^{2}\varphi }{d{\eta }^{2}}+\mathrm{Pr}[f+cg]\frac{d\theta }{d\eta }=0,$$

21 $$\frac{{d}^{2}\varphi }{d{\eta }^{2}}+ScSr\frac{{d}^{2}\theta }{d{\eta }^{2}}-Sc\gamma \varphi +Sc\left[f+cg\right]\frac{d\varphi }{d\eta }=0.$$

Boundary conditions for dimensionless equations are as below

22 $$\frac{{df}}{{d\eta }} = 1,f = f_{w} ,\frac{{dg}}{{d\eta }} = 1,g = \frac{{f_{w} }}{c},\theta = 1,\varphi = 1\;{\text{at}}\;\eta = 0,\frac{{df}}{{d\eta }} \to 0,\frac{{dg}}{{d\eta }} \to 0,\theta \to 0,\varphi \to 0\;{\text{at}}\;\eta \to \infty .$$

## Solution methodology

This section is presented to explain steps involved in implemented computational schemes (RK and Shooting) to find solution of governing physical problem. Since, we have attained coupled system of ODE’s in dimensionless form along with associated boundary conditions. The basic concept behind utilization of RK method is to transform BVP in to initial value problem by using following substitutions.

23 $$\begin{aligned} & f = y_{1} ,f^{\prime} = y_{2} ,f^{\prime\prime} = y_{3} ,g = y_{4} ,g^{\prime} = y_{5} ,g^{\prime\prime} = y_{6} ,\theta = y_{7} ,\theta ^{\prime} = y_{8} ,\theta ^{\prime\prime} = y_{8}^{\prime } ,\varphi = y_{9} , \\ & \varphi ^{\prime} = y_{{10}} ,\varphi ^{\prime\prime} = y_{{10}}^{\prime } . \\ \end{aligned}$$

After substituting variables demarcated in Eq. (23) the governing constitutive equation in the form of IVP is represented as below

24 $$\begin{gathered} y_{1}^{\prime } = y_{2} ,y_{2}^{\prime } = y_{3} , \hfill \\ y_{3}^{\prime } = \frac{{A_{2} (1 - \phi )^{{2.5}} }}{{A_{1} }}\left[ {(y_{2} )^{2} - \left( {y_{1} + cy_{4} } \right)y_{3} } \right], \hfill \\ y_{4}^{\prime } = y_{5} ,y_{5}^{\prime } = y_{6} , \hfill \\ y_{6}^{\prime } = \frac{{A_{2} \left( {1 - \phi } \right)^{{2.5}} }}{{A_{1} }}\left[ {c(y_{5} )^{2} - \left( {y_{1} + cy_{4} } \right)y_{6} } \right], \hfill \\ y_{7}^{\prime } = y_{8} , \hfill \\ y_{8}^{\prime } = \frac{{A_{3} }}{{A_{3} - ScSrDfPrA_{4} \left( {1 - \phi } \right)^{{2.5}} }}\left( {Sc\gamma y_{7} - Sc\left( {y_{1} + cy_{4} } \right)y_{8} + ScSrPrA_{4} \frac{{\left( {1 - \phi } \right)^{{2.5}} }}{{A_{3} }}\left( {y_{1} + cy_{4} } \right)y_{{10}} } \right), \hfill \\ y_{9}^{\prime } = y_{{10}} , \hfill \\ y_{{10}}^{\prime } = \frac{{A_{4} (1 - \phi )^{{2.5}} }}{{A_{3} }}\left[ {\frac{{ - DfPrA_{3} }}{{A_{3} - ScSrDfPrA_{4} \left( {1 - \phi } \right)^{{2.5}} }}\left( {Sc\gamma y_{7} - Sc\left( {y_{1} + cy_{4} } \right)y_{8} + ScSrPrA_{4} \frac{{\left( {1 - \phi } \right)^{{2.5}} }}{{A_{3} }}\left( {y_{1} + cy_{4} } \right)y_{{10}} } \right) - {\text{Pr}}(y_{1} + cy_{4} )y_{{10}} )} \right]. \hfill \\ \end{gathered}$$

The transformed constraints are as follows

25 $$\begin{aligned} & y_{1} \left( 0 \right) = f_{w} ,y_{2} \left( 0 \right) = 1,y_{4} \left( 0 \right) = \frac{{f_{w} }}{c},y_{5} \left( 0 \right) = 1,y_{7} \left( 0 \right) = 1,y_{9} \left( 0 \right) = 1\;{\text{and}} \\ & y_{2} \left( \infty \right) = 0,y_{5} \left( \infty \right) = 0,y_{7} \left( \infty \right) = 0,y_{9} \left( \infty \right) = 0. \\ \end{aligned}$$

Since the above scheme has eight first order equations, then to compute result with RK-integration pattern we required 10 initial conditions. But in Eq. (25) six initial conditions are available and four are missing. Thus, before preliminary solution procedure favorable initial estimate for ${y}_{3}\left(0\right), {y}_{6}\left(0\right), {y}_{8}\left(0\right), {y}_{10}(0)$ are selected. After choosing random values of ${\eta }_{\infty }$ at 2.5 with ${y}_{3}\left(0\right), {y}_{6}\left(0\right), {y}_{8}\left(0\right), {y}_{10}(0)$ are assigned to be − 1.

The comparison of result for limiting case was established at various value of $Pr$ when $\phi \frac{{A}_{1}}{{A}_{2}}=\phi \frac{{A}_{3}}{{A}_{4}}=1, {f}_{w}=0.3$ and $c=0.3$. As seen from Table 1, there is a good agreement with published work of T. Elnaqeeb et al. ^56^ for limiting case. Thermophysical features of nanoparticles cane be seen in Table 2.

**Table 1:** Comparative analysis of ${-\theta }^{^{\prime}}(0)$ with Elnaqeeb et al.^56^ for a limiting case.

Pr	Elnaqeeb et al.^56^	Present results
0.07	0.0655	0.0656
0.2	0.1690	0.1691
0.7	0.4530	0.4539
2	0.9113	0.9144
7	1.8954	1.8953

**Table 2:** Thermophysical features (density, thermal conductivity, specific heat) of base fluid and nanoparticles.

Nanoparticles and base fluid	$\rho$ (kg m^−3^)	$k$ (w m^−1^ K^−1^)	${c}_{p}$ (JK^−1^ kg^−1^)	Nanoparticles shapes
Water	997.1	0.613	4179	
Ternary carbon nanotubes	2100	3007.4	410	Spherical
Hybrid graphene	2200	5000	790	Cylindrical
Nanofluid 1 aluminium oxide	3970	40	765	Platelet
Ternary copper oxide	6500	20	535.6	Spherical
Hybrid copper	8933	400	385	Cylindrical
Nanofluid 2 silver	10,500	429	235	Platelet

The skin friction factors along x and y direction in dimensional form are represented as

26 $${C}_{fx}=\frac{{\mu }_{hnf}}{{\rho }_{bf}{a}^{2}{x}^{2}}\frac{\partial u}{\partial z}\left|z=0\right., {C}_{gy}=\frac{{\mu }_{hnf}}{{\rho }_{bf}{a}^{2}{y}^{2}}\frac{\partial T}{\partial z}\left|z=0\right..$$

The skin friction factors along x and y direction in dimensionless form are represented as

27 $$f^{\prime\prime}\left( 0 \right) = \frac{{\left( {1 - \phi } \right)^{2.5} C_{fx} \surd R_{{e_{x} }} }}{{A_{1} }},g^{\prime\prime}\left( 0 \right) = \frac{{\left( {1 - \phi } \right)^{2.5} C_{gy} \surd R_{{e_{y} }} }}{{cA_{1} }}.$$

The computation of the heat transfer rate (Nusselt number) along x-axis and along y-axis in dimensional form are given as,

28 $$N{u}_{x}=\frac{-x{k}_{hnf}}{{k}_{bf}({T}_{w}-{T}_{\infty })}\frac{\partial T}{\partial z}\left|z=0\right.,N{u}_{y}=\frac{-y{k}_{hnf}}{{k}_{bf}({T}_{w}-{T}_{\infty })}\frac{\partial T}{\partial z}\left|z=0\right. .$$

The computation of the heat transfer rate (Nusselt number) along x-axis and along y-axis in dimensionless form are given as,

29 $$\frac{{(1-\phi )}^{2.5}{N}_{{u}_{x}}}{{A}_{3}\surd {R}_{{e}_{x}}}=\frac{{(1-\phi )}^{2.5}{N}_{{u}_{y}}}{{A}_{3}\surd {R}_{{e}_{y}}}=-{\theta }^{^{\prime}}\left(0\right).$$

The Sherwood number in dimensional form is given as

30 $$Sh=\frac{{j}_{w}x}{D({C}_{w}-{C}_{\infty })}, \mathrm{where},{j}_{w}=-D\left(\frac{\partial C}{\partial z}\right) \mathrm{at} \ z=0.$$

The Sherwood number in dimensionless form as under

31 $$-{\varphi }^{^{\prime}}\left(0\right)=\frac{Sh}{\sqrt{R{e}_{x}}}.$$

## Results and discussion

**Figure 2:**
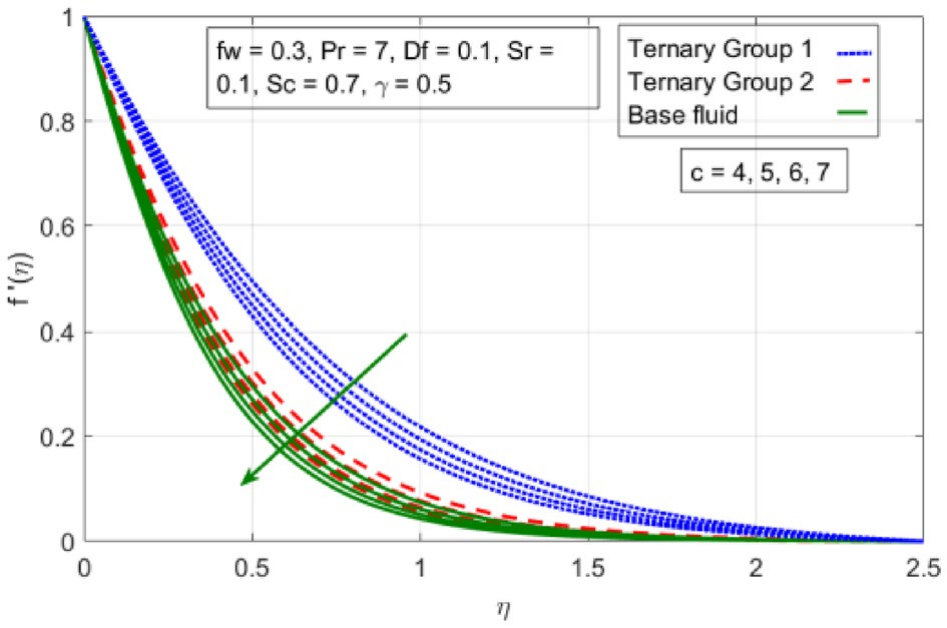
Variation in velocity profile against stretching ratio (c) along x-axis.

**Figure 3:**
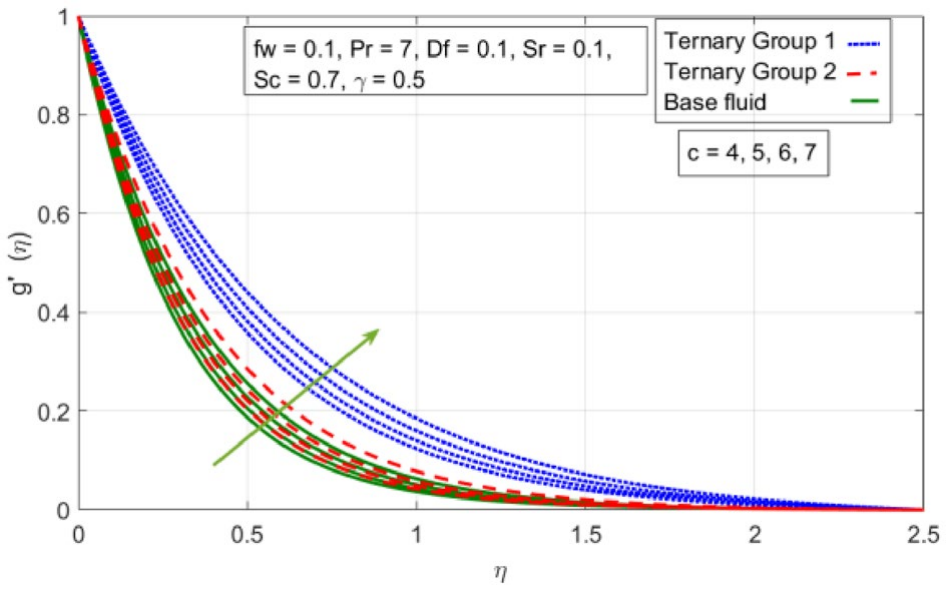
Variation in velocity profile against stretching ratio (c) along y-axis.

**Figure 4:**
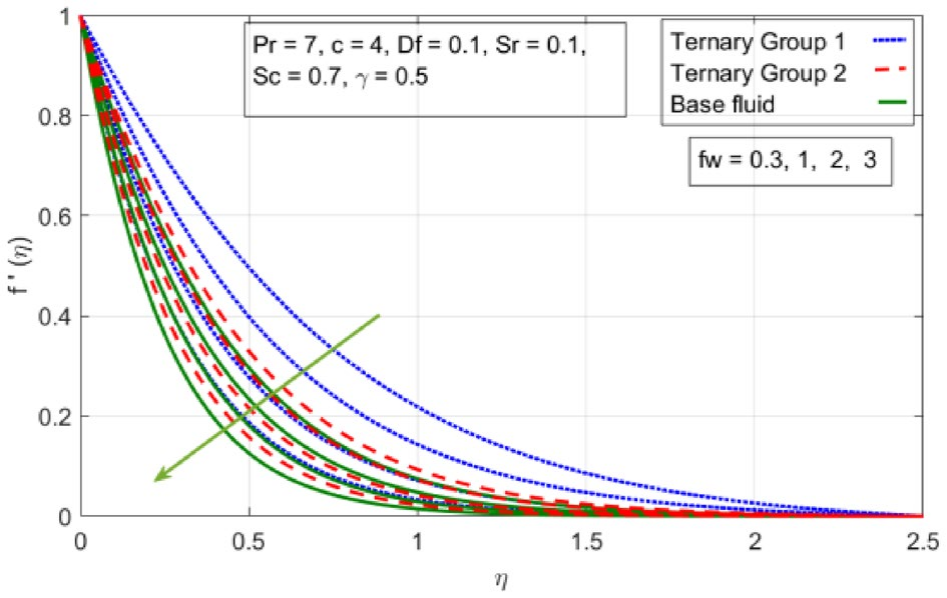
Variation in velocity profile against suction velocity ${(f}_{w})$ along x-axis.

**Figure 5:**
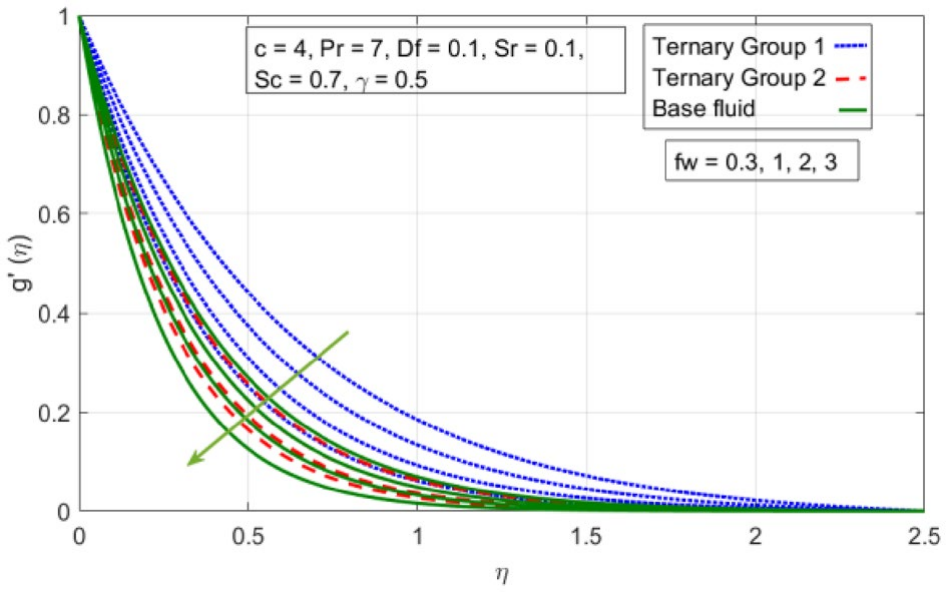
Variation in velocity profile against suction velocity ${(f}_{w})$ along y-axis.

**Figure 6:**
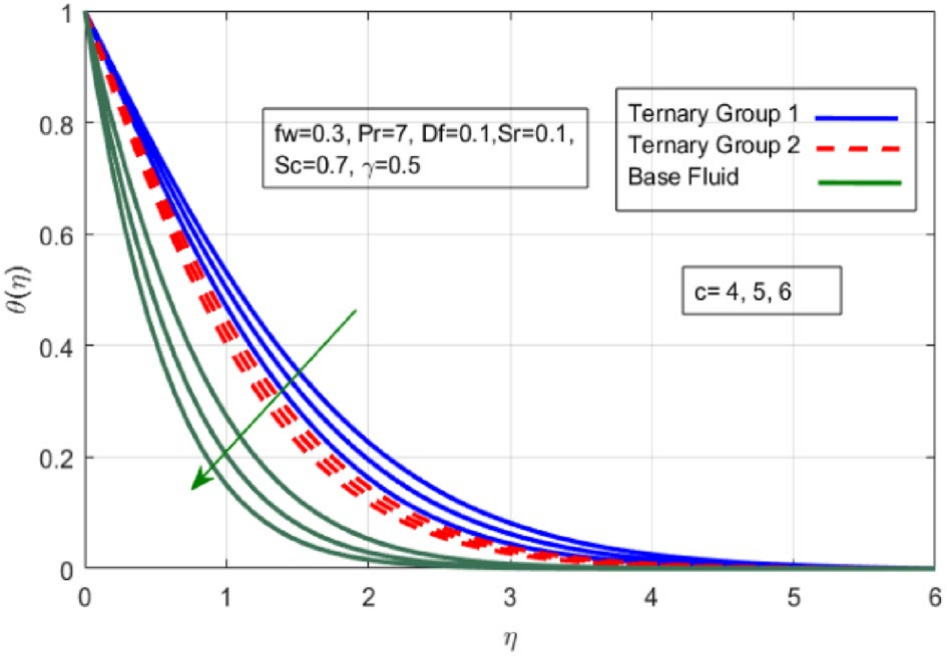
Variation in temperature against stretching ratio (c).

**Figure 7:**
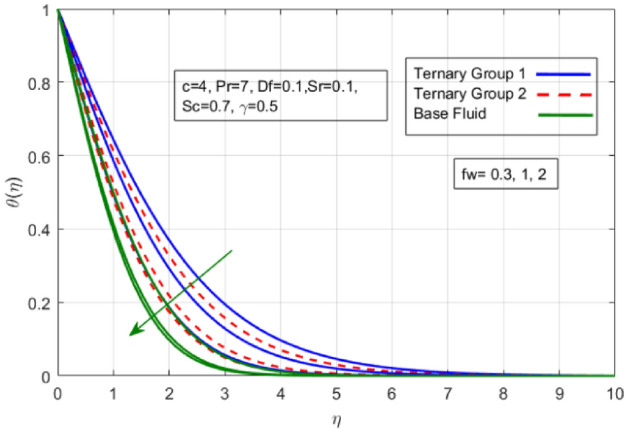
Variation in temperature against suction velocity ${(f}_{w})$.

**Figure 8:**
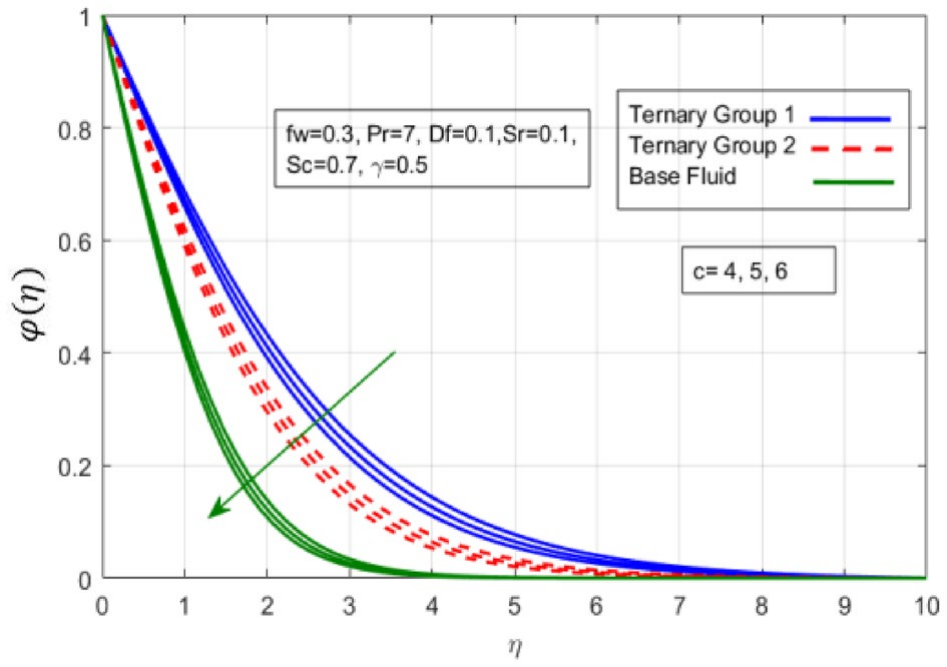
Variation in concentration against stretching ratio (c).

**Figure 9:**
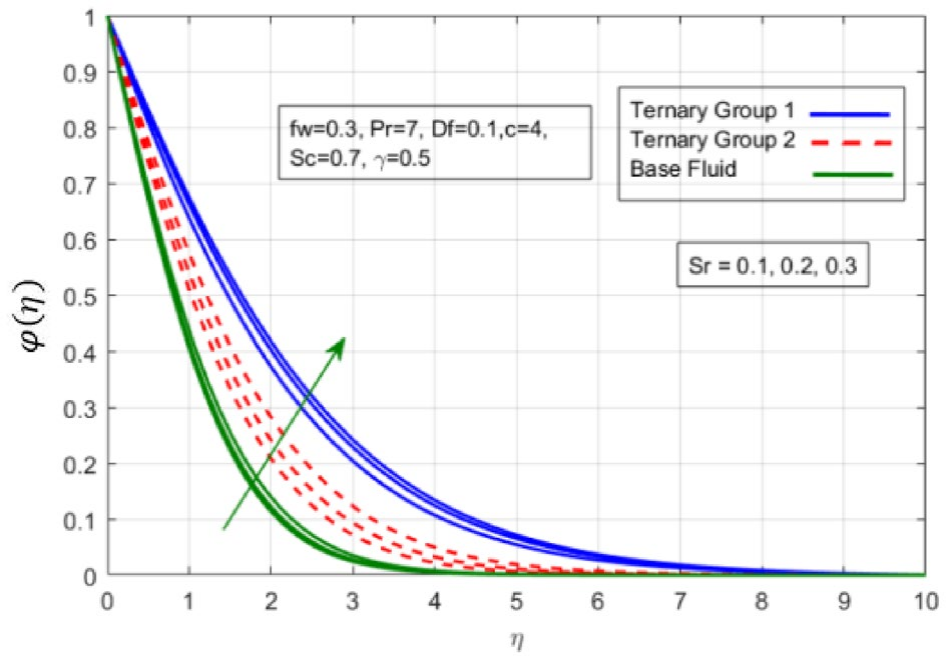
Variation in concentration against Soret number (Sr).

**Figure 10:**
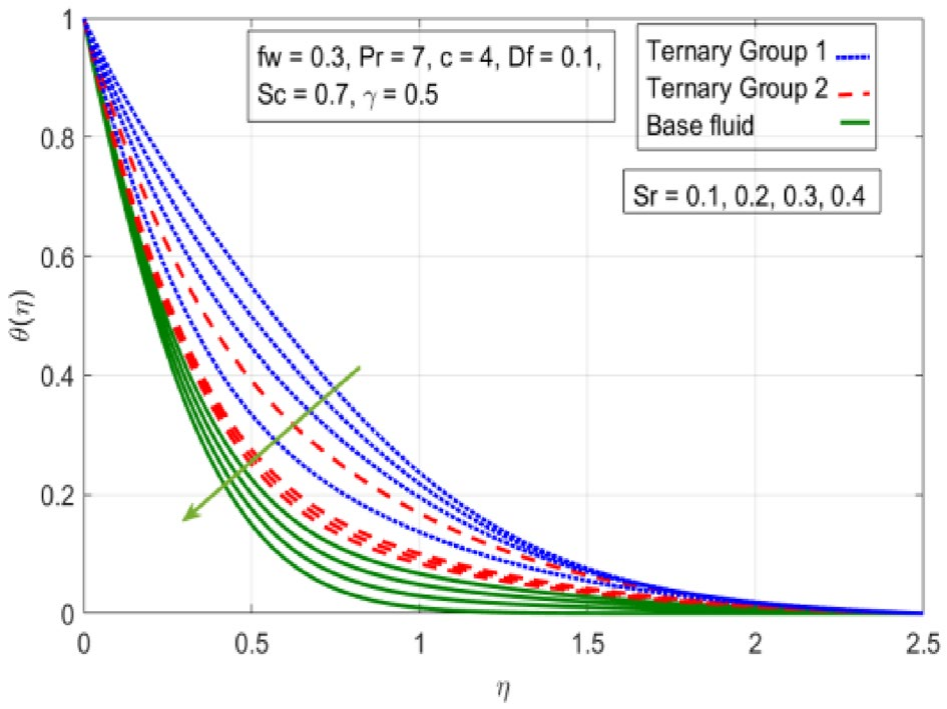
Variation in temperature against Soret number (Sr).

**Figure 11:**
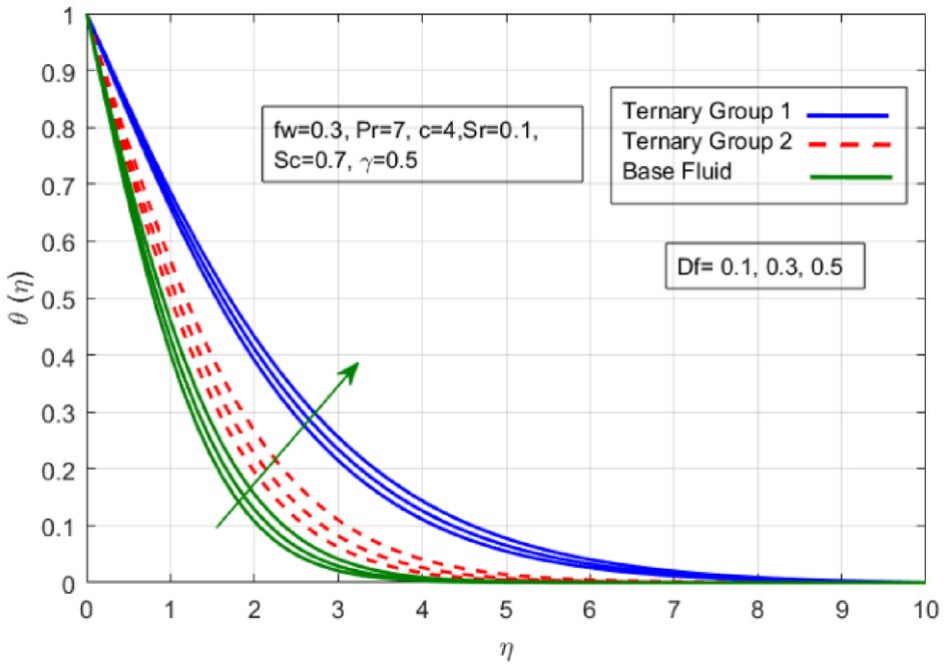
Variation in temperature against Dufour number $(Df)$.

**Figure 12:**
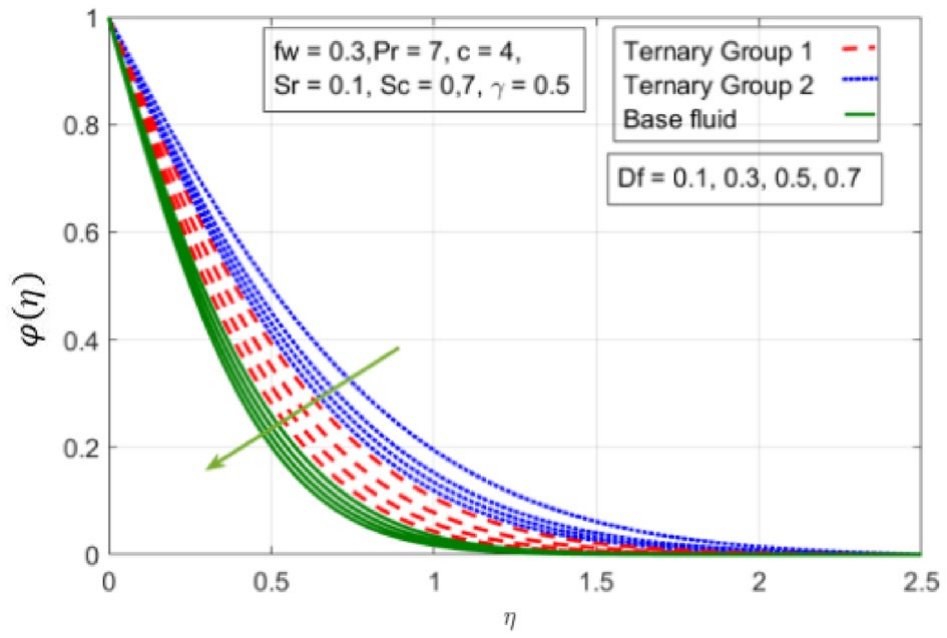
Variation in concentration against Dufour number $(Df)$.

**Figure 13:**
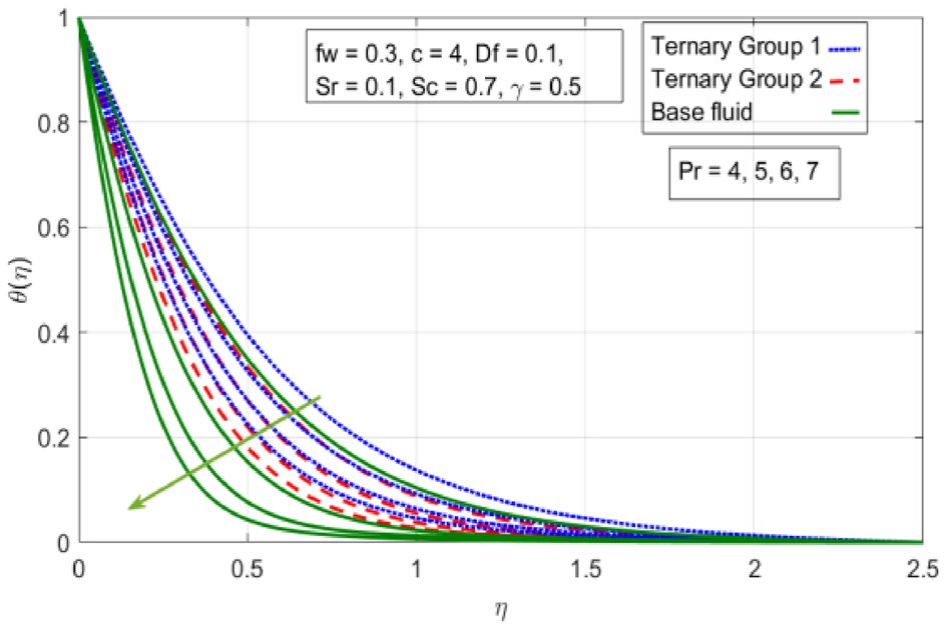
Variation in temperature against Prandtl number (Pr).

**Figure 14:**
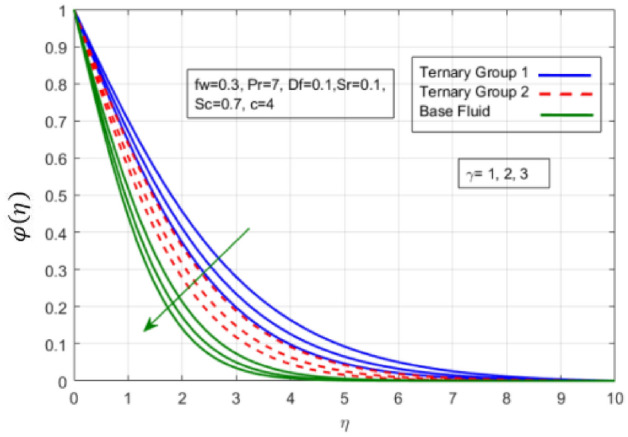
Variation in concentration against chemical reaction (γ).

**Figure 15:**
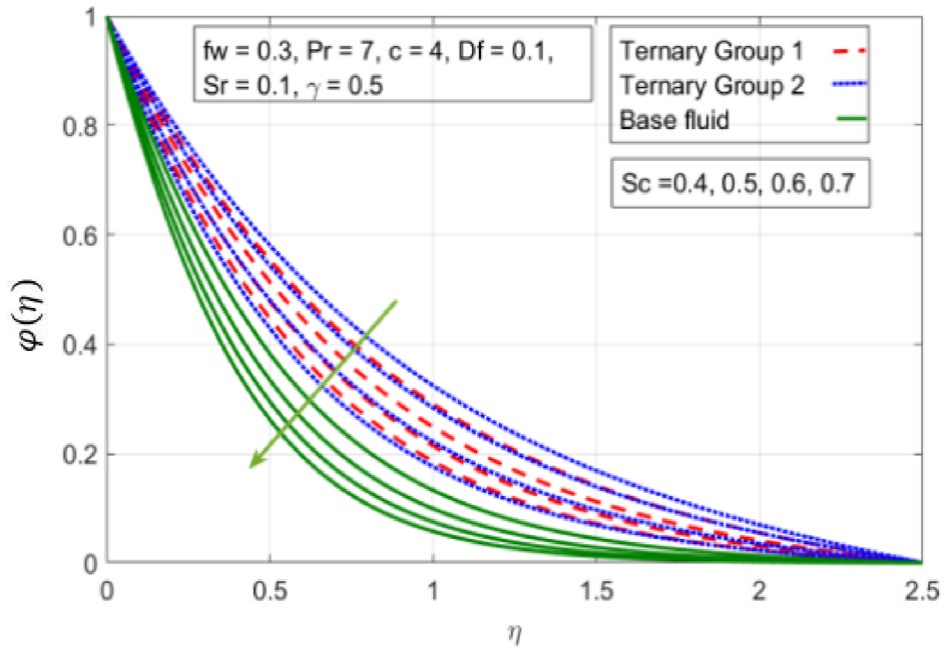
Variation in concentration against Schmidt number (Sc).

**Figure 16:**
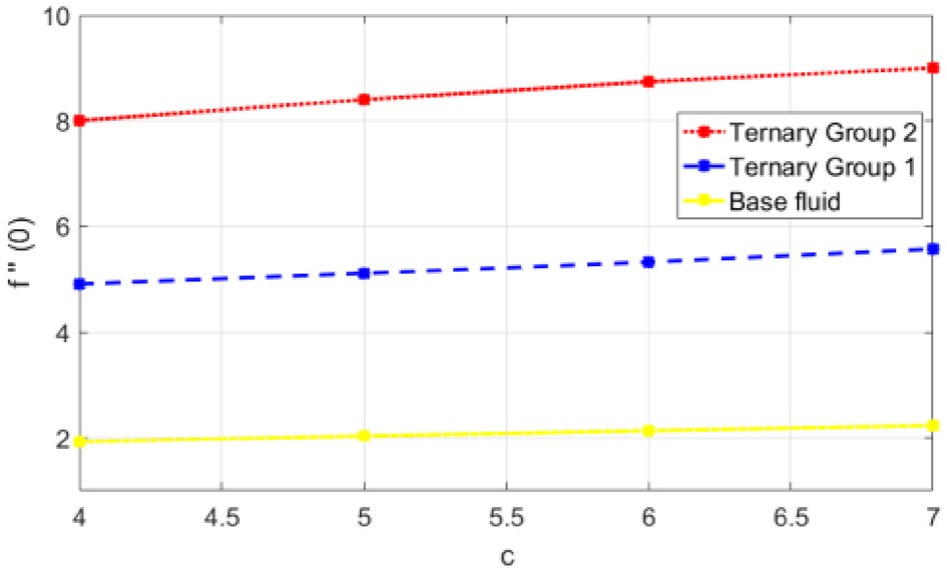
Variation in skin friction against stretching ratio (c) along x-direction.

**Figure 17:**
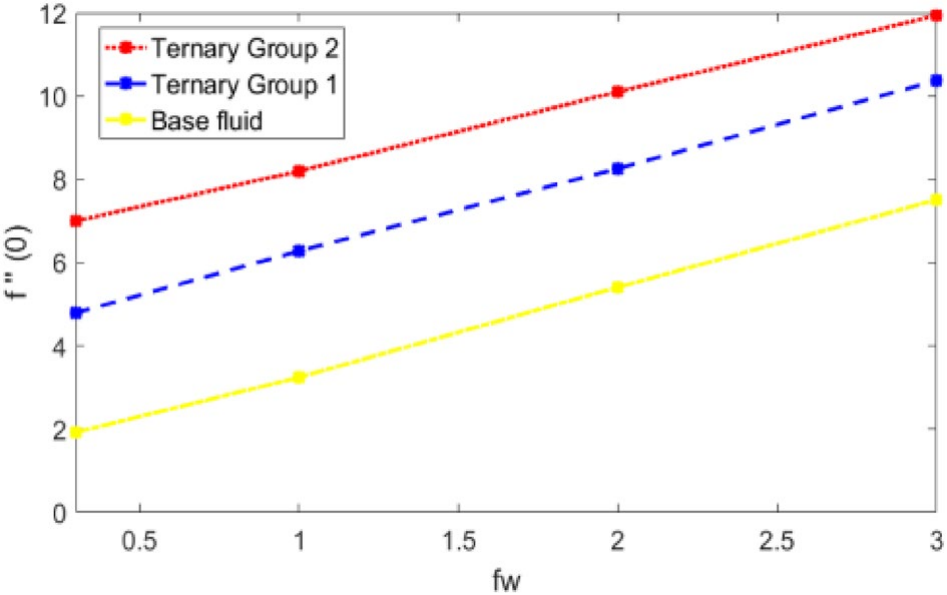
Skin friction coefficient along x-axis against suction velocity (fw).

**Figure 18:**
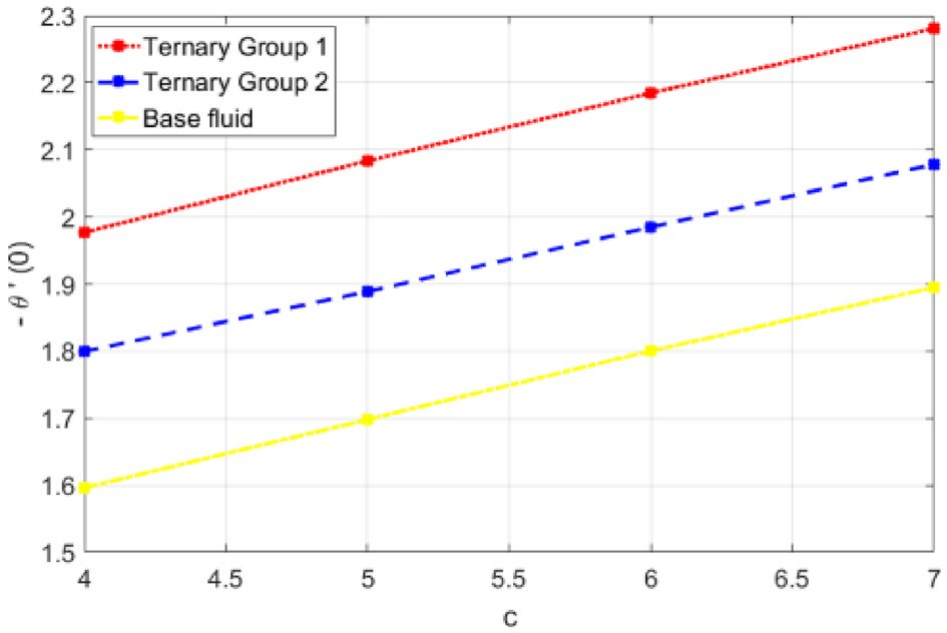
Deviation in Nusselt number against stretching ratio (c).

**Figure 19:**
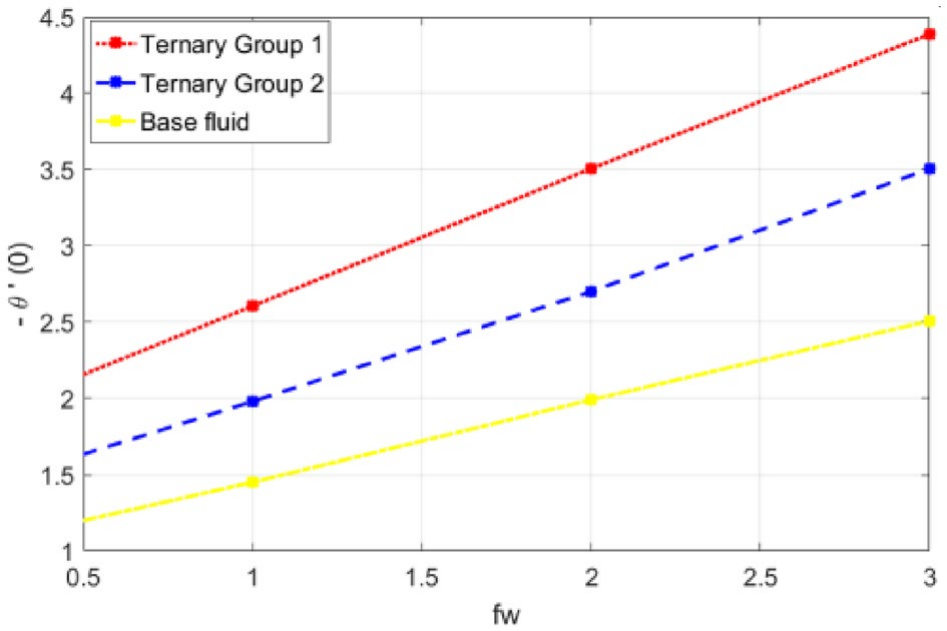
Deviation in Nusselt number against suction velocity ${(f}_{w})$.

**Figure 20:**
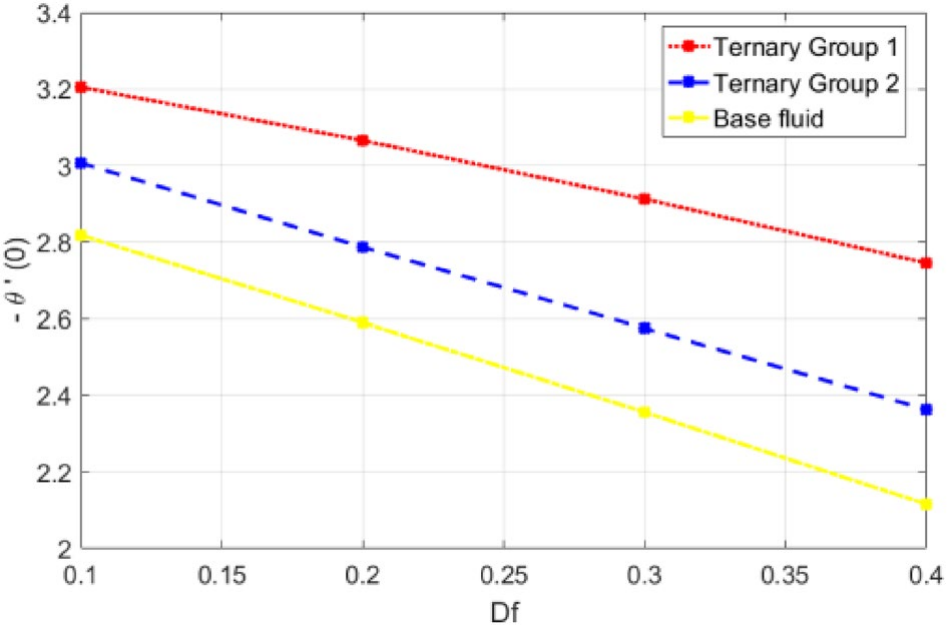
Deviation in Nusselt number against Dufour number $(Df)$.

**Figure 21:**
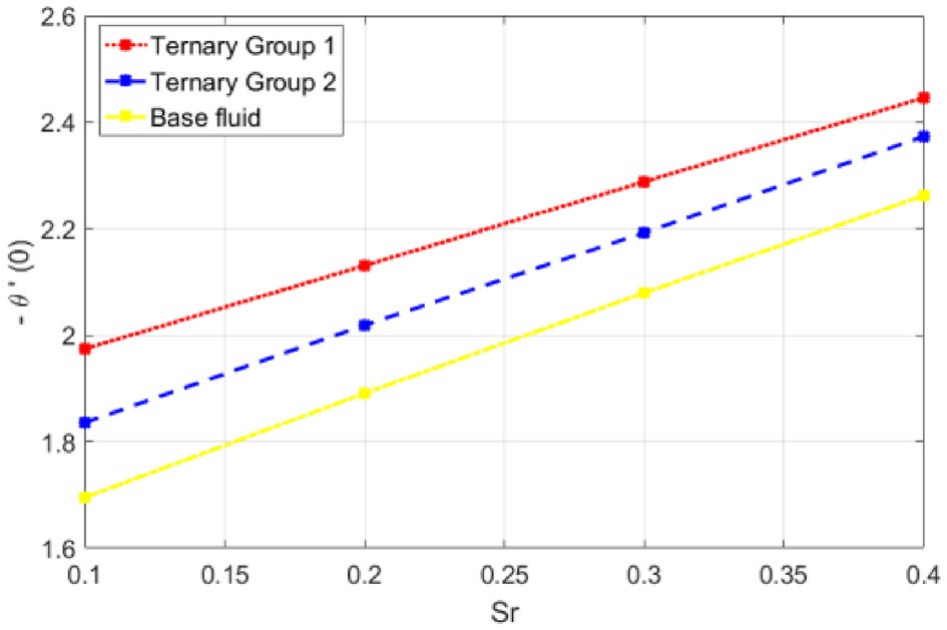
Deviation in Nusselt number against Soret number (Sr).

**Figure 22:**
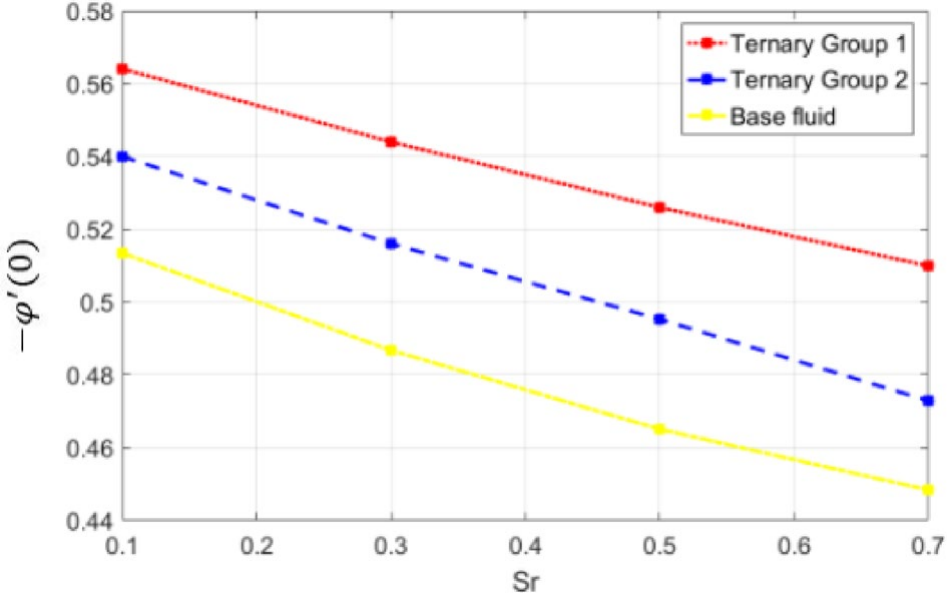
Deviation in Sherwood number against Sr number (Sr).

**Figure 23:**
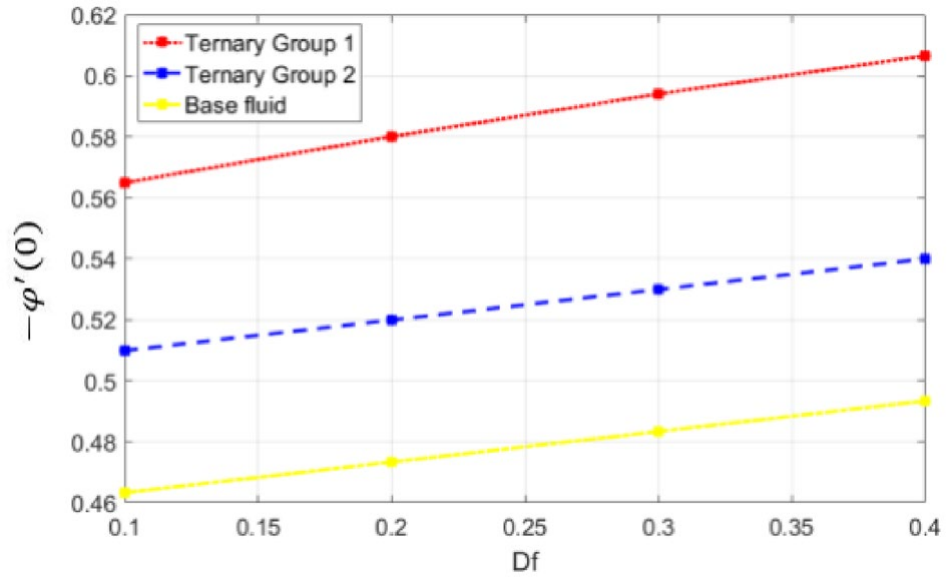
Deviation in Sherwood number against Dufour $(Df)$.

This portion is presented to explain the outcome of dissimilar factors on velocity, concentration and temperature fields. For this purpose initially mathematical formulation of the problem is manifested in form of dimensionless ODEs along with associated boundary conditions. Afterwards, numerical simulations are executed by implementing shooting method with Runge–Kutta scheme of order 4. Influences of involved physical parameters in comparative manner for two diversified compositions of ternary nanoparticles on associated distributions are divulged in Figs. 2, 3, 4, 5, 6, 7, 8, 9, 10, 11, 12, 13, 14 and 15. The capacities of engineering interest like skin friction coefficients along $(x,y)$ directions, Nusselt and Sherwood numbers against stretching ratio *(c)* suction velocity ${(f}_{w})$ parameters and $Df, Sr$ effects are shown in Figs. 16, 17, 18, 19, 20, 21, 22 and 23.

Figures 2, 3, 4 and 5 is sketched to explicate the impact of stretching ratio parameters (c) and suction velocity ${(f}_{w})$ on horizontal velocity *f*′(*η*) and vertical velocity components *g*′(*η*). The influence of stretching ratio (c) on horizontal and vertical components of velocity by fixing ${f}_{w}=0.3, Pr=4, {\phi }_{1}={\phi }_{2}={\phi }_{3}=0.15, Pr=7, Df=0.1, Sr=0.1, Sc=0.7, \gamma =0.5$ is addressed in Figs. 2 and 3. Here, two groups of ternary nanoparticles with small densities (Carbon nanotubes, Graphene and Aluminium oxide) and large densities (Copper oxide, Copper and Silver) are taken into account and water as base fluid is divulged. From Figs. 2 and 3 it is seen that due to uplift in stretching ratio (c) velocity profile along x-direction (*f*′(*η*)) diminishes. It is due the fact that by using (c) stretching rate along x-axis direction decreases because $c=\frac{b}{a}$. In addition, it is also noticed that velocity distribution for ternary group 2 containing particles (Copper oxide, Copper and Silver) is higher than the ternary group 1 (Carbon nanotubes, Graphene and Aluminium oxide) due to high density effect. Figure 3 explicitly reveals the significant effect of stretching ratio parameter (c) on vertical component of velocity *g*′(*η*) by fixing $fw=0.3, Pr=7$, ${\phi }_{1}={\phi }_{2}={\phi }_{3}=0.15, Df=0.1, Sr=0.1, Sc=0.7, \gamma =0.5$. The increasing trend in *g*′(*n*) against (c) is observed by taking two different groups of ternary nanoparticles with high and low densities in to account. It is because of the fact that by enhancing (c) the stretching rate along y-direction increments due to which momentum of fluid elevates along y-axis. This reason is also justified by mathematical relation i.e., c = $\frac{b}{a}$. In addition it is seen that velocity excessive in case of ternary group 1 than ternary group 2 due to smaller densities. From boundary layer point of view it is seen that in Figs. 2 and 3 it is achieved at $\eta =2.5$.

The diminishing impact of suction parameter ${(f}_{w})$ on velocity distributions in $(x-,y-)$ directions when $c=4, Pr=7, Df=0.1, Sr=0.1, Sc=0.7, \gamma =0.5,$ ${\phi }_{1}={\phi }_{2}={\phi }_{3}=0.15$ on boundary layer flow of water containing nanoparticles with lower densities (Carbon nanotubes, Graphene and Aluminium oxide) and larger densities (Copper oxide, Copper and Silver). It is depicted in Figs. 4 and 5 that by varying ${(f}_{w})$ in range of $0.3\le fw\le 3$ momentum of fluid decelerates. This behavior is justified by the magnitude of wall velocity given i.e. $fw=\frac{-Zw}{\sqrt{a{\vartheta }_{bf}}}$. From relation it is seen that by using ${(f}_{w})$ rate of stretching along x-direction decreases. Deviation in temperature distribution against stretching ratio parameter (c) and suction velocity ${(f}_{w})$ is noticed in Figs. 6 and 7. Here, stretching ratio parameter *(c)* is varied between $4\le c\le 7$ and other parameters like ${f}_{w}=0.3, Pr = 7, Df = 0.1, Sr = 0.1, Sc = 0.7,$ and $\gamma =0.5$ and suction velocity ${(f}_{w})$ $c = 4, Pr = 7, Df = 0.1, Sr = 0.1, Sc = 0.7$, and $\gamma =0.5$ are fixed. In addition, temperature distribution ($\theta (\eta )$) evaluates for higher density ternary particles (Copper oxide, Copper and Silver) and lower density ternary particles (Carbon nanotubes, Graphene and Aluminium oxide) and base fluid water. It is found that temperature of fluid decays with uplift in *(c)* and ${(f}_{w})$. This happens due to the fact that in *(c)* causes intensification in colder fluid as compared to hotter fluid region and reduces the ambient thermal potential. It is worthwhile to note that temperature of base fluid without adding ternary nanoparticles is lower than in the presence of particle. Whereas, the temperature in case of ternary group 1 (Carbon nanotubes, Graphene and Aluminium oxide) with lower densities is higher than the ternary group 2 (Copper oxide, Copper and Silver) with high densities. Figure 8 exhibits effect of stretching ratio *(c)* on concentration distribution. It is found that by increasing *(c)* concentration profile decrements. It is because of the fact that by increasing *(c)* stretching rate of surface over which fluid is located moves with accelerated rate due to which velocity of fluid along y-direction enhance whereas along x-direction decays. So, overall velocity of fluid uplift and concentration of fluid increases. Influence of Soret effect $(Sr)$ on concentration distribution for water based ternary nanofluid containing particles of higher densities (Copper oxide, Copper and Silver) and lower densities (Carbon nanotubes, Graphene and Aluminium oxide). Here, the other involved physical parameters like $fw=0.3, Pr=7, c=4, Df=0.1, Sr=0.1, Sc=0.7$, ${\phi }_{1}={\phi }_{2}={\phi }_{3}=0.15, \gamma =0.5$ are kept constants. It is seen in Fig. 9 by enhancing (Sr) on concentration distribution and associates boundary layer viscosity increment. It is because of the realities that by increasing $(Sr)$ then concentration gradient between wall and ambient fluid decreases due to which particle accumulate and concentrates. This logic is mathematically explained by relative existing i.e. $Sr=\frac{D{K}_{T}({T}_{w}-{T}_{\infty })}{{T}_{m}\upsilon ({C}_{w}-{C}_{\infty })}$. In addition, it is also observed that by uplifting $(Sr)$ the concentration field for particles possessing less densities ternary group 1 is lower than ternary group 2. Temperature distribution against Soret parameter $(Sr)$ is plotted in Fig. 10. It is perceived that impact of $(Sr)$ on temperature distribution is differing to that of $(Df)$. Effectiveness of Dufour number $(Df)$ in temperature distribution is delineated in Fig. 11. Again two compositions of nanoparticles are formed i.e. ternary group 1 (carbon nanotubes, Graphene and Aluminum oxide) containing particles of low densities and ternary group 2 (Copper oxide, Copper and Silver) are particles of higher densities. It is manifested that by rising $(Df)$ temperature of fluid elevates due to the fact that viscosity of fluid decreases and particles takes momentum and average temperature of fluid enhances. Figure 12 discusses the influence of Dufour parameter $(Df)$ on concentration distribution for water base liquid containing two diversified groups of ternary particles with low densities (Carbon nanotubes, Graphene and Aluminium oxide) and large densities (Copper oxide, Copper and Silver) by fixing $fw=0.3, Pr=7, c=4, Sr=0.1, Sc=0.7,$ γ = 0.5. It is seen that by uplifting magnitude of $(Df)$ concentration profile depreciates. It is because of the reason that by increasing $(Df)$ concentration difference between particles located at wall and at infinity enhances. Due to this generation of diffusion potential particles moves for range of higher to lower concentration of fluid decays. Decrementing trend in temperature against Prandtl number *(Pr)* is displayed in Fig. 13. It is because by enhancing *(Pr)* thermal diffusion of fluid decreases due to which temperature distribution show decreasing behavior. Variation in $\varphi$ ($\eta$) against chemical reaction parameter (γ) is addressed in Fig. 14. Here, positive magnitude of (γ) is assumed which shows the impact of constructive chemical reaction. In addition, to highlight optimization in behavior of flow field (γ) is varied between $1\le \gamma \le 4$ and other parameters $fw=0.3, Pr=7, c=4, Df=0.1, Sr=0.1, Sc=0.7$ are fixed. It is represented that by enhancing (γ) the module of base liquid (water) get polarized and diffusion of particles take place due to which concentration of fluid deseeds. Imprint of Schmidt number *(Sc)* on $\varphi (\eta )$ is manipulated in Fig. 15. Schmidt number is an important physical quantity in problem where mass transfer is to investigate. It is an analogue of Prandtl number like the (Pr) effect on temperature field similar decreasing influence of *(Sc)* on concentration distribution is revealed. It is because of fact that by increasing Schmidt number *(Sc)* viscous diffusion rises due to which particles moves extensively and convective potential rises. Figure 16 provides visualization about the influence of stretching ratio parameters on shear stress along x-direction. Here, *(c)* is chooses between 4 and 7 and behavior of wall dray in both direction for the case of two different group of ternary particles with higher and lower densities. Elevation in dray force exerted because with increase in *(c)* stretching rate along x-direction uplift and surface provide resistance to the flow. In addition, it is divulged that skin friction factor in case of addition of ternary nanoparticle with large densities depicts high magnitude. Figure 17 identifies the effect of suction velocity $(fw)$ on shear stress proportional to friction along x-direction by adding two diversified groups of ternary particles in base fluid (water). Since, by increasing ${(f}_{w})$ the quantity of fluid is drained out and effectiveness of friction force exerted by surface enhances due to which shear stress momentum. In addition, skin friction forces dominant for ternary group 2 due to presence of nanoparticles with higher densities. Heat transfer rate at all the stages of stretching ratio (c) and suction velocity ${(f}_{w})$ is proportional to Nusselt number across the dynamic of water and two groups of nanoparticles of smaller and larger densities which are shown in Figs. 18 and 19. Change in heat flux coefficient $(\frac{Sh}{\sqrt{Rex}}, \frac{Nux}{\sqrt{Rex}})$ against Dufour number $(Df)$ is shown in Fig. 20. Depreciation in Nusselt number is found against $(Df)$. Since, the mathematical relation of $Df=\frac{{DK}_{T}({C}_{w}-{C}_{\infty })}{{C}_{s}{C}_{p}({T}_{w}-{T}_{\infty })\upsilon }$ it is clear that $Df\propto \frac{1}{({T}_{w}-{T}_{\infty })}$. So by increasing (Df) temperature gradient and thermal potential decays due to which heat flux reduction. It is also observed that temperature behavior on Soret number (Sr) is totally inverse to Dufour number $(Df)$ that Soret number increases resulting the decrease in temperature shown in Fig. 21. The effect of Soret number (Sr) and Dufour Number on Sherwood number it clearly pointed that Dufour number $(Df)$ increases their values while inverse to Soret number *(Sr)* are shown in Figs. 22 and 23.

Comparative scrutiny of the importance of stretching ratio (c) and suction velocity $(fw)$. Performance of ternary nanoparticles by taking skin friction coefficient in x-direction and by fixing Pr, Df, Sr, Sc and γ is presented in the Table 3. Variation in skin friction factor along x-direction (− f″ (0)) against stretching ratio (c) and suction velocity $(fw)$ for ternary group 1 containing particles of low densities (Carbon nanotubes, Graphene and Aluminium oxide) and for ternary group 2 containing particles of large densities (Copper oxide, Copper and Silver) is enumerated in Table 3. It is observed that by increasing (c) and ${(f}_{w})$ magnitude of skin friction coefficient increments. To analyze optimize change in the mentioned engineering quantity wide range of stretching ratio (c) i.e. $4\le c\le 7$ and wall suction (fw) i.e. $0.3\le fw\le 3$ is assumed. The reason behind this behavior is that by increasing (c) the stretching rate along x-direction deseeds due to which velocity of fluid along x-direction diminishes and influence of wall dray forces increasing mounts. In similar fashion the influence of ${(f}_{w})$ increasing on $(cfx/\sqrt{Rex}$) is employed by the reason that with uplift in ${(f}_{w})$ the fluid is doomed out from the surface and velocity of base fluid (water) decreases and the influence of friction force mounts. It is worthy to mention that skin friction for the case of ternary group 2 (Copper oxide, Copper and Silver) is higher than from ternary group 1 (Carbon nanotubes, Graphene and Aluminum oxide) due to presence of dense particles in group.

**Table 3:** Comparison of skin friction coefficient (x-direction) against suction velocity (fw) and stretching ratio (c) for ternary group 1 and 2.

${f}_{w}$	c	− f″(0) Group 1	− f″(0) Group 2	− f″(0) Water
0.3		4.793713	7.004012	1.926347
1		6.280130	8.200457	3.244545
2		8.259938	10.106197	5.409734
3		10.374522	11.941125	7.509934
	4	4.793713	8.094012	1.926347
	5	5.065711	8.541748	2.034218
	6	5.322419	8.960170	2.135162
	7	5.566191	9.354310	2.230361

Table 4 portrays the variation in suction velocity ${(f}_{w})$, stretching ratio (c), Prandtl number (Pr), Dufour number $(Df)$, Soret number (Sr), Schmidt number (Sc) and chemical reaction on heat flux coefficient is divulged. Here, again two different groups of composition of ternary nanoparticles are considered namely ternary group 1 (Carbon nanotubes, Graphene and Aluminium oxide) and ternary group 2 (Copper oxide, Copper and Silver). It is illustrated that by uplift in suction velocity ${(f}_{w})$, stretching ratio (c), Prandtl number (Pr), flux increases. Whereas, contrary aspects of Dufour number (Df), Schmidt number (Sc) and chemical reaction (γ) on wall heat flux coefficient is revealed. The reason behind increment is (− θ′(0)) against involved physical parameters is collectively explained in a view that by increasing the parameter the thermal convective potential rises due to which temperature gradient in flow domain between cold and hot region rises and heat uplifts. Specifically traditional increasing effect of (Pr) temperature gradient is found.

**Table 4:** Comparative analysis of significance of ($fw, c, Pr, Df, Sr, Sc$, γ) on Nusselt number (− θ′ (0)).

${f}_{w}$	c	Pr	Df	Sr	Sc	γ	− θ′(0) Group 1	− θ′(0) Group 2	− θ′(0) Water
0.3							1.496240	1.976837	1.099484
1							1.980516	2.606210	1.451556
2							2.701051	3.506087	1.991129
3							3.505590	4.387691	2.508402
	4						1.699484	1.976837	1.696240
	5						1.768793	2.083591	1.817979
	6						1.834822	2.184669	1.930289
	7						1.898407	2.281061	2.035027
		1					1.699484	1.986737	1.396240
		2					2.455216	2.936568	2.117482
		3					3.236275	3.851409	2.891780
		4					3.994828	4.692680	3.579094
			0.1				2.936568	3.994828	2.817482
			0.2				2.746527	3.655576	2.590015
			0.3				2.555406	3.312303	2.356230
			0.4				2.363197	2.964937	2.115820
				0.1			1.705960	1.974317	1.695219
				0.3			1.938704	2.130610	1.890729
				0.5			2.262488	2.287971	2.079499
				0.7			2.573043	2.605782	2.262266
					0.7	1	1.696240	1.976837	1.699484
					0.8		1.695219	1.974317	1.695960
					0.9		1.694653	1.972131	1.692616
					1		1.694460	1.970266	1.689454
						2	1.673986	1.954287	1.674148
						3	1.654747	1.934276	1.653157
						4	1.637557	1.916183	1.633695

It is noticed in Table 5 that by increasing $(Df)$ mass flux increases whereas by increasing (Sr) on (− *ϕ*′(0)) deceeds. If these behaviors are justified by the fact that $(Df)$ has direct relation with concentration differences whereas Soret number (Sr) has inverse relation with concentration difference (${C}_{w}-{C}_{\infty }$). So by increasing (Df) the mass difference potential rises between particles concentration at wall and at ambient surface whereas opposite trend is seen against (Sr). Subsequently, it is seen that concentration flux in case of ternary group 1 (Carbon nanotubes, Graphene and Aluminium oxide) is more that group 2 containing particles of higher densities.

**Table 5:** Effectiveness of Dufour number $(Df)$ and Soret number (Sr) on mass flux coefficient.

$Df$	Sr	− φ′(0) Group 1	− φ′(0) Group 2	− φ′(0) Water
0.1		0.489923	0.473374	0.584976
0.2		0.499943	0.483389	0.595173
0.3		0.509962	0.493405	0.605370
0.4		0.519982	0.503420	0.615567
	0.1	0.539982	0.513420	0.565407
	0.3	0.517628	0.486745	0.544011
	0.5	0.495257	0.465056	0.525999
	0.7	0.472871	0.448355	0.509928

## Conclusion

Current effort is made to investigate Dufour and Soret effects on 3D flow of water based hybrid nanofluid by inserting ternary hybrid nano composition of low and higher densities namely ternary group 1 (Carbon nanotube, Graphene and Aluminium oxide) and ternary group 2 (Copper oxide, Copper and Silver). Mathematical formulation of problem manifested in the form of PDEs containing thermo physical features of ternary particles. Afterwards, similarity transformations provided by Ramesh et al.^56^ is employed to transform in to ODEs. Numerical simulations by implementing Shooting scheme with Runge–Kutta technique of order 4 are computed. Impact of flow concerning factor on velocity, temperature and concentration field are interpreted through graphical visualization in comparative manner for two ternary groups. Capacities of engineering interest like skin friction, heat flux and mass flux coefficients are determined through groups and tables. The key outcomes are enlisted as follows.

- Velocity and temperature distributions exceeds in case of ternary group 1 containing particles of low densities than the ternary group 2 comprising of particles with large densities.
- In view of concentration distribution for ternary hybrid nanoparticles with high densities is more than with low densities.
- Against stretching ratio factor (c) velocity distribution (${f}^{^{\prime}}(\eta )$) along x-direction decreases whereas along y-direction (${g}^{^{\prime}}(\eta )$) increases.
- Temperature and concentration profiles of fluid down surges against stretching ratio parameter $(c).$
- Velocity and temperature distributions against suction velocity shows decrementing behavior whereas concentration of fluid enhances.
- Temperature profile against Dufour and Soret number in which temperature decreases against Soret number $(Sr)$ and increases against Dufour number $(Df)$ and against concentration the Dufour and Soret number have opposite behavior to temperature.
- Temperature profile against Prandtl number $(Pr)$ and concentration profiles against Schmidt number $(Sc)$ and chemical reaction (γ) all of these shows the decreasing behavior.
- Depreciation in Nusselt number is found against increasing values of Dufour number $(Df)$.
- Sherwood number increases by increasing Dufour number $(Df),$ while inverse behaviour can be seen in case of Soret number $(Sr).$

## Data Availability

All the data contain within the manuscript.

## Abbreviations

$a$: Stretching rate for the motion along x-direction
$b$: Stretching rate for the motion along y-direction
$c$: Ratio off stretching rate
$C{f}_{x}$: Local skin friction coefficients (friction in x-direction)
$C{f}_{y}$: Local skin friction coefficients (friction in y-direction)
$\frac{df}{d\eta }$: Dimensionless horizontal velocity of the motion in x-Direction
$\frac{dg}{d\eta }$: Dimensionless horizontal velocity of the motion in y-Direction
$f(\eta )$: Dimensionless vertical velocity of the motion in x-direction
$g(\eta )$: Dimensionless vertical velocity of the motion in y-direction
${f}_{w}$: Dimensionless suction velocity
${k}_{hnf}$: Thermal conductivity of the ternary hybrid fluid
${k}_{bf}$: Thermal conductivity of the base fluid
$$\eta$$: Dimensionless distance
${Nu}_{x}$: Nusselt number (heat transfer along x-direction)
${Nu}_{y}$: Nusselt number (heat transfer along y-direction)
${\rho }_{bf}$: Density of the base fluid (kg m^−^^3^)
${({\rho c}_{p})}_{hnf}$: Heat Capacity of the ternary hybrid nanofluid (J °C^−^^1^ kg)
${\rho }_{hnf}$: Density of the ternary hybrid nanofluid
$\phi$: Volume fraction of the ternary hybrid nanofluid
${\phi }_{1}$: Volume/amount of spherical nanoparticles
${\phi }_{2}$: Volume/amount of cylindrical nanoparticles
${\phi }_{3}$: Volume/amount of platelet nanoparticles
$$\theta (\eta )$$: Dimensionless temperature
$$Re$$: Reynold number
$T$: Dimensional temperature of the ternary hybrid nanofluid (°C)
${T}_{w}$: Dimensional wall temperature
${T}_{\infty }$: Temperature of the fluid far away from the wall
${\mu }_{1}\mathrm{and} {K}_{1}$: Viscosity and thermal conductivity of spherical based nanofluid
${\mu }_{3}\mathrm{and} {K}_{3}$: Viscosity and thermal conductivity of platelet based nanofluid
${\mu }_{hnf}$: Dynamic viscosity of the ternary hybrid nanofluid (kg m^−1^ s^−1^)
$u$: Dimensional velocity of the motion along x-direction (m s^−1^)
${u}_{w}$: Stretching velocity for the motion along x-direction
$v$: Dimensional velocity of the motion along y-direction
${v}_{w}$: Stretching velocity for the motion along y-direction
$w$: Dimensional velocity of the motion along z-direction
${w}_{w}$: Dimensional suction velocity
$x$: Dimensional horizontal distance parallel to the motion
$y$: Dimensional horizontal distance perpendicular to the motion
$z$: Dimensional vertical distance perpendicular to x-axis and y-axis
${z}_{w}$: Dimensional Suction rate
